# Developmental malformations resulting from high-dose maternal tamoxifen exposure in the mouse

**DOI:** 10.1371/journal.pone.0256299

**Published:** 2021-08-17

**Authors:** Miranda R. Sun, Austin C. Steward, Emma A. Sweet, Alexander A. Martin, Robert J. Lipinski

**Affiliations:** Department of Comparative Biosciences, School of Veterinary Medicine, University of Wisconsin-Madison, Madison, WI, United States of America; University College London, UNITED KINGDOM

## Abstract

Tamoxifen is an estrogen receptor (ER) ligand with widespread use in clinical and basic research settings. Beyond its application in treating ER-positive cancer, tamoxifen has been co-opted into a powerful approach for temporal-specific genetic alteration. The use of tamoxifen-inducible Cre-recombinase mouse models to examine genetic, molecular, and cellular mechanisms of development and disease is now prevalent in biomedical research. Understanding off-target effects of tamoxifen will inform its use in both clinical and basic research applications. Here, we show that prenatal tamoxifen exposure can cause structural birth defects in the mouse. Administration of a single 200 mg/kg tamoxifen dose to pregnant wildtype C57BL/6J mice at gestational day 9.75 caused cleft palate and limb malformations in the fetuses, including posterior digit duplication, reduction, or fusion. These malformations were highly penetrant and consistent across independent chemical manufacturers. As opposed to 200 mg/kg, a single dose of 50 mg/kg tamoxifen at the same developmental stage did not result in overt structural malformations. Demonstrating that prenatal tamoxifen exposure at a specific time point causes dose-dependent developmental abnormalities, these findings argue for more considerate application of tamoxifen in Cre-inducible systems and further investigation of tamoxifen’s mechanisms of action.

## Introduction

Tamoxifen is the oldest synthetic selective estrogen receptor (ER) modulator and is widely used in both clinical and basic research applications. Included in the World Health Organization list of essential medicines, tamoxifen is used to treat individuals with ER-positive breast cancer. Tamoxifen is also increasingly applied in biomedical research as part of powerful genetic recombination systems that leverage a fusion protein of Cre recombinase and a mutated ligand binding domain of the human ER (ERT). Upon tamoxifen binding to the ERT, nuclear translocation of the fusion protein facilitates Cre-mediated excision of loxP-flanked sequences in DNA [1]. Tamoxifen-inducible systems that allow temporally controlled genetic recombination are used for gene deletion, gene overexpression, and lineage tracing. This toolkit is applied to examine genetic, molecular, and cellular mechanisms, including those governing embryonic and postnatal development.

Although tamoxifen-inducible Cre models are widely employed to achieve temporal-specific gene alteration, developmental effects independent of Cre recombination have been suggested in multiple reports [2–10]. Animal model studies have shown that *in utero* tamoxifen exposure can cause mammary tumors, proliferative uterine lesions, oviduct hyperplasia, and impaired oocyte differentiation in offspring [5–7]. However, some reports also suggest that tamoxifen may have additional impacts on development that are not linked to ER signaling and endocrine disruption. Two recent reports described structural malformations in mouse embryos following maternal tamoxifen treatment [8, 11], while published human case reports document congenital anomalies associated with tamoxifen treatment during pregnancy, including craniofacial and limb defects [9, 10].

Here, we examined the impact of acute prenatal tamoxifen exposure on embryonic development in wildtype C57BL/6J mice. Our findings demonstrate that prenatal tamoxifen exposure causes structural limb and craniofacial malformations in a dose-dependent manner and suggest a previously unrecognized mechanism of action that may have significant implications for its use in clinical and basic research settings.

## Results

### Tamoxifen-induced malformations

**Fig 1:**
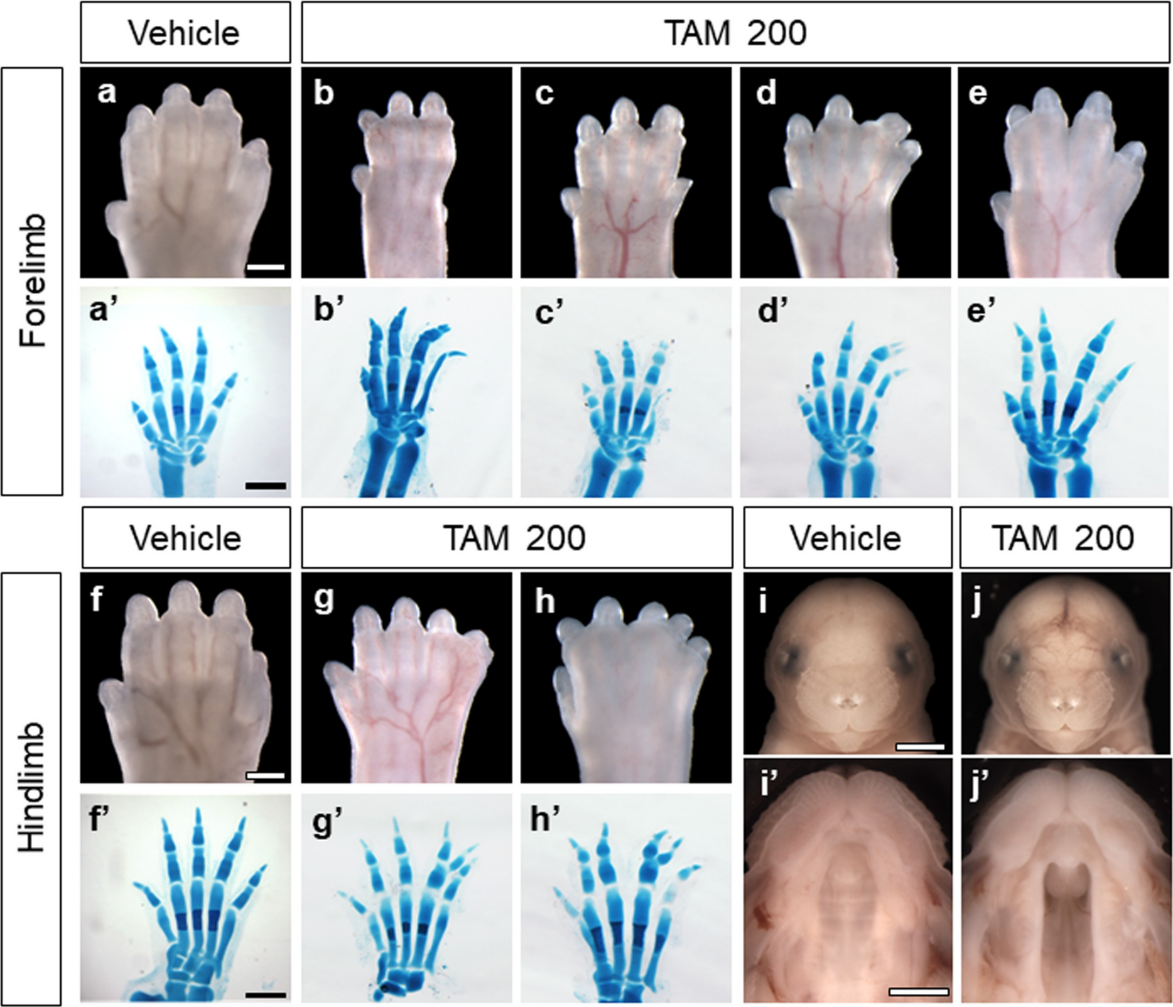
Tamoxifen-induced limb and craniofacial malformations. Along with representative vehicle controls of forelimb (a), hindlimb (f), and palate (i’), examples of malformations in forelimbs (b-e), hindlimbs (g, h), and palate (j’) of 200 mg/kg tamoxifen-treated animals are shown at GD17. Bone and cartilage staining of tamoxifen-treated animals revealed underlying skeletal abnormalities compared to controls (a’-h’). In the forelimbs of animals with reduction malformations (b, c), dysmorphology in both the metacarpals and phalanges of the 5^th^ digit are apparent. Additionally, the distal-most phalanx of the 4^th^ digit appears to be duplicated in these animals (b’, c’). An animal with a forelimb duplication malformation (d) exhibited a replication of the distal phalanx of the 4^th^ digit (d’). In hindlimbs of animals with duplication malformations (g, h), staining revealed replications of the distal and middle phalanges of the 4^th^ digit (g’, h’). In an animal with a non-reduction/duplication phenotype (e), no overt skeletal abnormalities are apparent (e’). Secondary cleft palate (j’) was also observed in 200 mg/kg tamoxifen-treated animals. TAM, tamoxifen. Scale bars in a, a’, f, and f’: 0.5 mm; Scale bar in i: 2.0 mm; Scale bar in i’: 1.0 mm.

Timed pregnant wildtype C57BL/6J mice were administered a single dose of 50 mg/kg or 200 mg/kg tamoxifen (Sigma) or vehicle alone by IP injection at gestational day (GD)9.75. Dams were euthanized at GD17, and uteri were carefully inspected. The number of live fetuses and resorptions in each litter as well as mean crown-rump length were calculated (Table 1). No statistically significant differences in average number of surviving fetuses, average number of resorptions, or average fetal crown-rump length were detected between treatment groups. No overt structural malformations were observed in either the vehicle-exposed control group (Fig 1A, 1A’, 1F, 1F’, 1I, 1I’) or in the 50 mg/kg tamoxifen-exposed group. In the 200 mg/kg tamoxifen dose group, limb malformations were observed including posterior digit reduction (Fig 1B and 1C), posterior digit duplication with or without fusion (Fig 1D, 1G and 1H), and other distal limb defects (Fig 1E). Bone and cartilage staining of animals with gross limb malformations further revealed abnormalities in the metacarpals and phalanges (Fig 1B’–1E’, 1G’ and 1h’). Inspection of the oral cavity also revealed cleft palate in the group exposed to 200 mg/kg tamoxifen (Fig 1J and 1J’) but not in the group exposed to 50 mg/kg tamoxifen.

**Table 1:** Descriptors of the wildtype C57BL/6J study population found in Fig 2.

Treatment (Distributor)	Litters Collected	Live Fetuses (Mean ± SD)	Resorptions (Mean ± SD)	Crown-rump Mean ± SD (mm)
**Vehicle**	9	52 (5.8 ± 1.91)	14 (1.56 ± 1.61)	16.63 ± 0.73
**TAM 50 mg/kg (Sigma)**	11	67 (6.1 ± 2.07)	13 (1.18 ± 1.40)	17.03 ± 1.24
**TAM 200 mg/kg (Sigma)**	21	93 (4.4 ± 2.85)	61 (2.90 ± 2.89)	16.80 ± 1.08
**TAM 200 mg/kg (Selleckchem)**	3	18 (6 ± 3.56)	1 (0.33 ± 0.47)	16.25 ± 1.12

Timed-pregnant wildtype C57BL/6J mice were administered the indicated doses of tamoxifen (TAM) at gestational day (GD)9.75 and inspected at GD17 for live fetuses and resorptions. One-way ANOVA was used to compare mean numbers of live fetuses, resorptions, and crown-rump lengths between vehicle- and TAM-treated groups. No statistically significant differences were detected across treatment groups. TAM, tamoxifen; SD, standard deviation.

**Fig 2:**
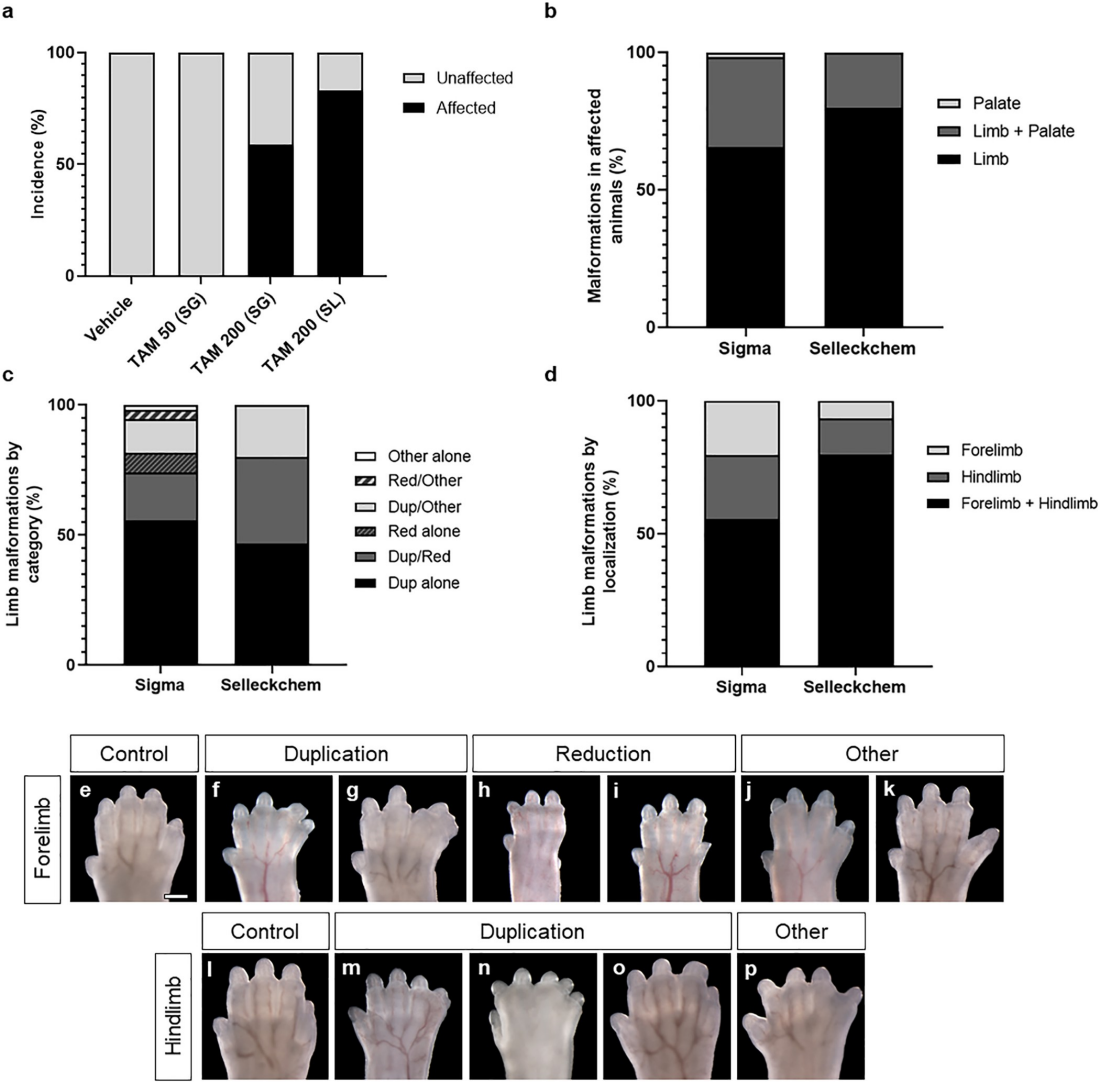
Incidence of tamoxifen-induced limb and craniofacial malformations. The incidence of malformations occurring in animals treated with tamoxifen or vehicle at GD9.75 are summarized (a-d). The percentage of animals displaying at least one structural malformation in each treatment group is shown (a). Structural malformations appeared in animals treated with 200 mg/kg Sigma or Selleckchem tamoxifen, and these affected animals displayed either limb malformations, craniofacial malformations, or co-occurring limb and craniofacial malformations (b). Except for a single animal exhibiting an isolated secondary cleft palate, all affected animals exhibited limb malformations (b). Therefore, limb abnormalities were further classified according to apparent dysmorphology (c) and limb-specific localization of the abnormality (d). Examples of limb morphology in vehicle controls and tamoxifen-treated animals (e-p). A duplication phenotype was assigned to limbs that exhibited an extra posterior digit with or without cutaneous fusion to adjacent digits in both forelimbs (f, g) and hindlimbs (m, n, o). Posterior digit reduction phenotypes were exclusive to forelimbs and included apparent absence of a 5^th^ digit (h), or a shortening of the 5^th^ digit (i). Non-reduction/duplication phenotypes included apparent hyperplasia of interdigital tissue between the 2^nd^ and 3^rd^ forelimb digits (j) and laterally displaced 5^th^ digits in forelimbs (k) and hindlimbs (p). Scale bar in a: 0.5 mm. TAM, tamoxifen; SG, Sigma; SL, Selleckchem; Dup, duplication; Red, reduction.

The incidence of tamoxifen-induced structural malformations as visualized by whole mount brightfield imaging is shown in Fig 2. In the 200 mg/kg treatment group, gross structural malformations were observed in 59.14% of fetuses collected (n = 55/93) with affected fetuses identified in 15/21 litters. The majority of affected fetuses exhibited limb malformations (n = 54/93) including apparent posterior digit duplication with or without fusion (n = 48/54), apparent posterior digit reduction (n = 16/54), or other defects (n = 10/54). Limb abnormality patterns within individually affected fetuses were diverse and included both reduction and duplication phenotypes in separate limbs (n = 10/54), duplication and non-reduction/duplication phenotypes in separate limbs (n = 8/54), and reduction and non-reduction/duplication phenotype in separate limbs (n = 2/54). Malformations were present in both hindlimbs and forelimbs (n = 30/54), restricted to forelimbs (n = 11/54), or restricted to hindlimbs (n = 13/54). Cleft palate was observed in 20.43% of fetuses (n = 19/93), with 18 of 19 fetuses with cleft palate also exhibiting limb malformations.

To test whether these outcomes were dependent upon chemical manufacturer, a parallel trial was conducted using tamoxifen supplied by an independent manufacturer and distributor (Selleckchem). No statistical differences in average number of surviving fetuses, average number of resorptions, or fetal crown-rump length were detected when comparing the 200 mg/kg Selleckchem tamoxifen group against the 200 mg/kg Sigma tamoxifen group and vehicle-exposed groups (Table 1). In the 200 mg/kg Selleckchem tamoxifen group, malformations were observed in 83.33% of fetuses (n = 15/18), with affected individuals identified in each of the 3 litters. All affected fetuses exhibited limb malformations, including posterior digit duplication with or without fusion (n = 15/15), posterior digit reduction (n = 5/15), or other defects (n = 3/15). All animals that exhibited a posterior digit reduction or other defect additionally exhibited a duplication malformation. In this tamoxifen (Selleckchem) cohort, most affected fetuses exhibited malformations affecting both the fore- and hindlimbs (n = 12/15), while a minority had only forelimb (n = 1/15) or only hindlimb abnormalities (n = 2/15). Cleft palate was 16.67% penetrant in these fetuses (n = 3/18) and always accompanied by limb malformations.

## Discussion

The tamoxifen/CreERT genetic recombination system is a powerful and widely used approach to examine molecular and cellular mechanisms of development and disease. However, off-target effects of tamoxifen treatment, including Cre toxicity [12], may confound interpretation of results from the use of these models [13]. Previous studies examining the potential of tamoxifen to affect developmental processes have focused upon outcomes related to endocrine modulation. Here, we demonstrate that *in utero* high-dose tamoxifen exposure results in developmental abnormalities in the absence of genetic recombination and causing structural malformations in wildtype C57BL/6J mice. Specifically, we found that prenatal tamoxifen exposure at a precise critical period of development results in malformations of the limbs and palate. These findings are consistent with several published reports of prenatal tamoxifen exposure. One clinical case report described limb (“club foot”) and craniofacial (micrognathia and cleft palate) malformations in a child exposed to tamoxifen during the first trimester [14]. Two recent reports noted structural malformations in mice exposed to tamoxifen *in utero*, including neural tube defects, cleft palate, and eye abnormalities [8, 11]. These observations suggest that tamoxifen has off-target effects that may impact its use in mouse model research and that usage of lower doses may be a strategy to mitigate these off-target effects.

Tamoxifen is used in CreERT transgenic models to trigger temporally specific Cre-recombinase activity in adult mice, neonates, and embryos and fetuses *in utero* via maternal administration. Across published studies, protocols for tamoxifen administration to pregnant dams in order to induce recombination in embryos vary considerably with respect to preparation, route, dose, duration of exposure, and co-administration with other drugs such as progesterone [15–39]. In this study, we chose a single IP administration of 50 or 200 mg/kg to encompass commonly utilized administration regimens [19, 26, 36–39]. We also tested whether developmental effects caused by tamoxifen were consistent across multiple suppliers of tamoxifen. While a high dose of tamoxifen from both suppliers caused structural malformations, there was a (not statistically significant) trend toward higher resorptions and lower malformation penetrance in the Sigma cohort and lower resorptions and higher malformation penetrance in the Selleckchem cohort. Whether the activity demonstrated here for tamoxifen administration is retained by other tamoxifen manufacturers, administration routes, drug preparations, or administration of tamoxifen metabolites that also activate ERT (*e*.*g*., 4-hydroxytamoxifen) is unclear and should be examined.

The observations reported herein follow two recent reports of structural malformations in wildtype mice following prenatal tamoxifen exposure. In one report, repeated tamoxifen exposure targeting slightly later critical periods of development (~GD10.5–13.5) was found to cause cleft palate [11]. In another report, embryos and fetuses from pregnant dams dosed with 10–200 mg/kg tamoxifen between GD5.5 and GD7.5 exhibited abnormal development and malformations, including neural tube defects, cleft palate, and eye abnormalities [8]. In this previous study, 8 out of 8 dams were reported to develop severe intrauterine hemorrhaging, and maternal toxicity was implicated as a causal or contributing factor to the observed developmental outcomes. The observations collected for the study described herein, however, are not consistent with a significant role for maternal toxicity in causing the malformations that followed targeted administration of tamoxifen. In our study cohort of wildtype C57BL/6J mice, a total of 11 dams were treated with 50 mg/kg tamoxifen and 24 dams were treated with 200 mg/kg tamoxifen at GD9.75. Signs of overt maternal toxicity were not observed, and no significant differences were detected in litter size at GD17, number of resorbs, or crown-rump lengths of fetuses (Table 1). Rather than a constellation of developmental outcomes, targeted tamoxifen exposure produced consistent and specific malformations that, to our knowledge, are not associated with maternal toxicity. While a role of maternal toxicity cannot be excluded, the observations reported herein better align with the premise that tamoxifen disrupts embryonic developmental through a currently unknown mechanism.

Tamoxifen’s biological effects are thought to be predominantly mediated through its binding to ERα and ERβ [40, 41]. To our knowledge, neither limb nor palate malformations have been reported to directly result from endocrine disruption and have not been reported in *Esr1* (ERα) or *Esr2* (ERβ) knockout mice [42, 43]. We performed preliminary experiments to examine the impact of *in utero* tamoxifen exposure in *Esr1*^-/-^ and *Esr2*^-/-^ mice (Jax strains 026176 and 004745, respectively) where timed-pregnant dams were administered 200 mg/kg tamoxifen (Sigma) at GD9.75, and structural malformations in offspring were examined at GD17 as in our wildtype cohort. While the sample size generated was not large enough for statistical analyses of malformation incidence across each genotype, limb malformations were observed in some *Esr1*^*-/-*^ and *Esr2*^*-/-*^ fetuses (S1 Fig and S1 and S2 Tables). A previous study also reported that mRNA expression of *Esr1* and *Esr2* was not detected in the limb during the critical period examined here for tamoxifen-induced limb malformations [44]. Together, these findings suggest that the mechanism by which tamoxifen exposure results in structural malformations may be independent of ER signaling, but determining whether ER or G protein coupled ER signaling plays any role in tamoxifen-induced structural malformations will require further investigation.

While thought to act predominantly through ER signaling, tamoxifen has previously been shown to affect processes such as proliferation, apoptosis, and angiogenesis through ER-independent mechanisms [45–54]. Additionally, tamoxifen or its metabolites have been reported to directly bind to off-target receptors such as aryl hydrocarbon receptor [55] and histamine, muscarinic, and dopamine receptors [53]. Previous studies have also reported that tamoxifen potently interferes with cholesterol synthesis *in vitro* and *in vivo* and that this activity is ER-independent [56–58]. Several signaling pathways are dependent upon cholesterol biosynthesis, including the Sonic hedgehog (Shh) signaling pathway. Covalent binding of cholesterol produces fully active SHH peptide that initiates signaling in effector cells through the Smoothened protein [59], the activity of which was recently also shown to be dependent upon cholesterol binding [60, 61]. Shh signaling plays multiple roles in development including craniofacial and limb morphogenesis, and disruption of this pathway at the same time point as the one used in this study has been shown to cause cleft palate and limb malformations [62], consistent with those reported herein to result from *in utero* tamoxifen exposure. This period during development encompasses major tissue patterning events, including limb and orofacial morphogenesis, and these processes may be particularly susceptible to chemical perturbation [63]. Future investigation into the mechanism(s) underlying the biological impacts of tamoxifen should benefit from demonstration herein that *in utero* tamoxifen exposure at a specific critical period in development results in specific malformations.

Here, we present a previously unreported action of tamoxifen that may have important implications for its use in both clinical and basic research settings. Demonstrating that a high dose of tamoxifen during a specific critical period in development causes structural malformations in the absence of genetic recombination suggests previously undescribed mechanisms for this widely used drug. We focused on structural malformations because they are novel, robust, and highly penetrant, but the importance of understanding whether and how tamoxifen acts through additional mechanisms is likely not limited to developmental contexts, as most developmental signaling pathways play roles in postnatal homeostasis, healing and repair, and disease processes. These findings, along with other reports illustrating off-target effects in utilization of CreERT models, support a more considerate use of these systems and interpretation of generated experimental results.

## Methods

### Materials

Tamoxifen used in these studies was obtained from two independent chemical distributors. Tamoxifen from MilliporeSigma (Catalog No. T5648, Lot No. WXBC7537V) was manufactured by AstraZeneca and had a supplier-stated purity of 99.00%. In this study, tamoxifen from MilliporeSigma is abbreviated as “Sigma”. Tamoxifen from Selleckchem (Catalog No. S1238, Lot No. S123802) was manufactured by Selleckchem and had a supplier-stated purity of 99.04%. Both vendors reported tamoxifen to have a solubility of 50 mg/ml in DMSO as well as a melting point between 96–98°C.

### Timed mouse mating

These studies were conducted in strict accordance with the recommendations in the *Guide for the Care and Use of Laboratory Animals* of the National Institute of Health. The protocol was approved by the University of Wisconsin School of Veterinary Medicine Institutional Animal Care and Use Committee (Protocol No. 13–081.0). Wildtype C57BL/6J (Strain No. 00664) mice were purchased from The Jackson Laboratory and housed under specific pathogen-free conditions in disposable, ventilated cages. Rooms were maintained at 22 ± 2°C and 30–70% humidity on a 12-h light, 12-h dark cycle. Mice were fed Irradiated Soy Protein-Free Extruded Rodent Diet (Catalog No. 2920x; Envigo Teklad Global) until day of plug, when dams received Irradiated Teklad Global 19% Protein Extruded Rodent Diet (Catalog No. 2919; Envigo Teklad Global). For timed matings, 1–2 nulliparous female mice were placed with a single male for 1–2 h and subsequently examined for the presence of copulation plugs as previously described [64]. The beginning of the mating period was designated as gestational day (GD)0.

### Tamoxifen exposure

Tamoxifen (Sigma or Selleckchem) was dissolved into solution with corn oil (Acros Organics). Aliquots were stored at 4°C and used within 3 weeks of preparation. Pregnant mice were administered an intraperitoneal (IP) injection of either 50 mg/kg tamoxifen, 200 mg/kg tamoxifen, or corn oil vehicle at GD9.75 ± 0.05.

### Dissection and imaging

Pregnant females were euthanized by CO_2_ asphyxiation followed by cervical dislocation at GD17 ± 2 h. Fetuses were placed in phosphate buffered saline on ice for no less than 30 min before dissection. Crown-rump measurements and initial gross phenotyping were conducted before fetal specimens were fixed in 10% formalin for at least 24 h before imaging. Images were taken of whole body, face, and palate using a MicroPublisher 5.0 camera connected to an Olympus SZX-10 stereomicroscope.

### Phenotypic assessment

Fetuses were assessed for gross malformations by thorough visual inspection. For each fetus, whole mount brightfield images were captured to document morphology, with a focus on the limbs and craniofacial regions. Limb malformations were grouped into one of three classes of overt distal limb abnormalities: apparent posterior digit duplication with or without fusion, apparent posterior digit reduction, and additional defects that do not exhibit overt digit reduction or duplication (Fig 2E–2P). Craniofacial malformations were characterized by the presence of secondary palate clefting. Animals were classified as affected by displaying at least one of these malformations.

### Bone & cartilage staining

Bone and cartilage staining was employed to reveal underlying skeletal abnormalities in animals classified as affected based upon careful scrutiny of light images. Following euthanasia, fetal specimens with overt structural malformations were skinned, eviscerated, and fixed in 95% or 100% ethanol for at least 3 d, then placed overnight in an alcian blue staining solution containing 8 ml of 100% ethanol, 10 ml of 100% glacial acetic acid, and 2 ml of 1% alcian blue in 3% acetic acid. They were then rinsed twice for 1 h and subsequently left overnight in 100% ethanol. Following clearing for 2 h in 1% potassium hydroxide, staining in 0.005% alizarin red in 2% potassium hydroxide for 4 h was performed. Fetuses were then rinsed in 2% potassium hydroxide once quickly, once for 1 h, and then left overnight in 1% potassium hydroxide. Finally, they were transferred to 1:3 solution of glycerol: 2% potassium hydroxide for 8 h and stored and imaged in a 1:1 solution of glycerol: 2% potassium hydroxide.

### Statistics

Graphpad Prism 8 was used for all statistical analyses. One-way analysis of variance (ANOVA) with Tukey’s post hoc test for multiple comparisons was used for analyses of litter sizes, resorptions, and crown-rump lengths. An alpha value of 0.05 was maintained for determination of significance for all experiments.

## Supporting information

S1 Fig Structural malformations in *Esr1* and *Esr2* animals. Representative examples of distal limb dysmorphology in hindlimbs of 200 mg/kg tamoxifen-treated animals (b, d, f, h) are shown along with vehicle controls (a, c, e, g). TAM 200, tamoxifen 200 mg/kg. Scale bars in a, e: 0.5 mm.
(TIF)

S1 Table Descriptors of the *Esr1* and *Esr2* study populations. Female *Esr1*^+/-^ mice were mated with male *Esr1*^+/-^ mice, and female *Esr2*^+/-^ mice were mated with either *Esr2*^+/-^ or *Esr2*^-/-^ male mice. Timed-pregnant dams were administered 200 mg/kg of tamoxifen at gestational day (GD)9.75. At GD17, dams were inspected for live fetuses and resorptions, and fetuses were measured for crown-rump length and genotyped from tail tissue. TAM, tamoxifen; SD, standard deviation.
(DOCX)

S2 Table Incidence of malformations by genotype in *Esr1* and *Esr2* animals. Incidence of limb malformations, cleft palate, or limb malformations or cleft palate are listed for each genotype and treatment group of the *Esr1* and *Esr2* study populations. TAM, tamoxifen.
(DOCX)
