# Supplementary figures and images for: Developmental malformations resulting from high-dose maternal tamoxifen exposure in the mouse
Source: PLoS One. 2021 Aug 17;16(8):e0256299. doi: 10.1371/journal.pone.0256299 (PMC8370643; doi:10.1371/journal.pone.0256299)

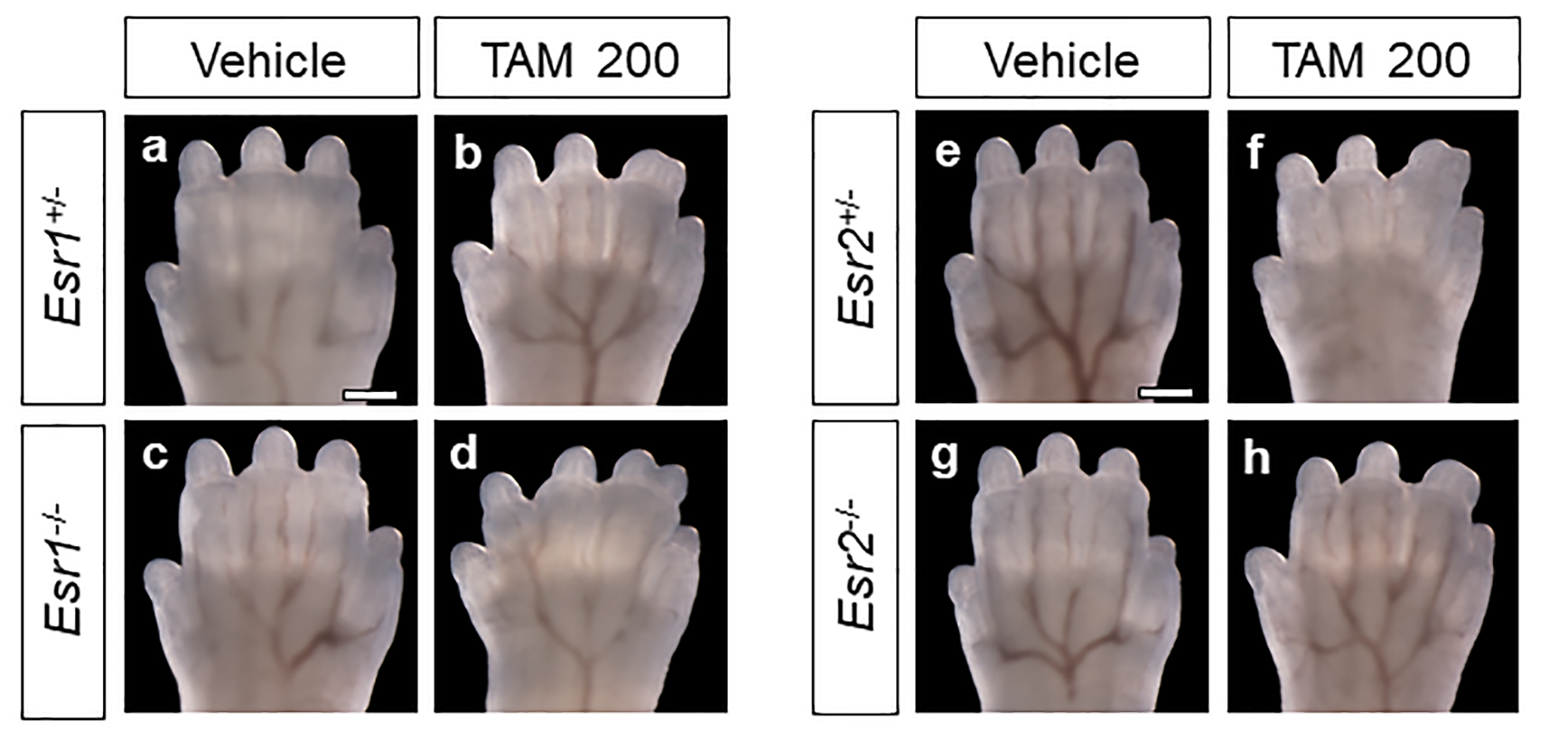

Supplement: S1 Fig — Representative examples of distal limb dysmorphology in hindlimbs of 200 mg/kg tamoxifen-treated animals (b, d, f, h) are shown along with vehicle controls (a, c, e, g). TAM 200, tamoxifen 200 mg/kg. Scale bars in a, e: 0.5 mm. (TIF) [file pone.0256299.s001.tif]
