# Supplementary material for: Developmental malformations resulting from high-dose maternal tamoxifen exposure in the mouse
Source: PLoS One. 2021 Aug 17;16(8):e0256299. doi: 10.1371/journal.pone.0256299 (PMC8370643; doi:10.1371/journal.pone.0256299)
Supplement: S1 Table — Female Esr1+/- mice were mated with male Esr1+/- mice, and female Esr2+/- mice were mated with either Esr2+/- or Esr2-/- male mice. Timed-pregnant dams were administered 200 mg/kg of tamoxifen at gestational day (GD)9.75. At GD17, dams were inspected for live fetuses and resorptions, and fetuses were measured for crown-rump length and genotyped from tail tissue. TAM, tamoxifen; SD, standard deviation. (DOCX) [file pone.0256299.s002.docx]

| Strain | Treatment (Distributor) | Litters Collected | Live Fetuses (Mean ± SD) | Resorptions (Mean ± SD) | +/+  Fetuses | +/- Fetuses | -/-  Fetuses | +/+ Fetuses Crown-rump Mean ± SD (mm) | +/- Fetuses Crown-rump Mean ± SD (mm) | -/- Fetuses Crown-rump Mean ± SD (mm) |
| --- | --- | --- | --- | --- | --- | --- | --- | --- | --- | --- |
| *Esr1* | Vehicle | 3 | 20 (6.7 ± 2.05) | 3 (1 ± 0) | 2 | 12 | 6 | 17 ± 0 | 17.33 ± 0.75 | 17.5 ± 0.41 |
| *Esr1* | TAM 200 mg/kg (Sigma) | 7 | 52 (7.4 ± 2.96) | 4 (0.57 ± 0.66) | 15 | 21 | 16 | 17.10 ± 1.10 | 16.69 ± 0.79 | 16.91 ± 0.92 |
| *Esr2* | Vehicle | 3 | 16 (5.3) | 9 (3 ± 1.92) | 0 | 9 | 7 | - | 16.67 ± 0.78 | 16.86 ± 0.52 |
| *Esr2* | TAM 200 mg/kg (Sigma) | 9 | 56 (6.2) | 22 (2.44 ± 2.18) | 2 | 22 | 32 | 18.25 ± 0.75 | 16.5 ± 1.30 | 16.31 ± 1.32 |
