# Supplementary material for: Developmental malformations resulting from high-dose maternal tamoxifen exposure in the mouse
Source: PLoS One. 2021 Aug 17;16(8):e0256299. doi: 10.1371/journal.pone.0256299 (PMC8370643; doi:10.1371/journal.pone.0256299)
Supplement: S2 Table — Incidence of limb malformations, cleft palate, or limb malformations or cleft palate are listed for each genotype and treatment group of the Esr1 and Esr2 study populations. TAM, tamoxifen. (DOCX) [file pone.0256299.s003.docx]

| Strain | Treatment (Distributor) | Genotype | Total Fetuses | Limb | | Palate | | Limb + Palate | |
| --- | --- | --- | --- | --- | --- | --- | --- | --- | --- |
|  |  |  |  | Affected | Unaffected | Affected | Unaffected | Affected | Unaffected |
| *Esr1* | Vehicle | +/+ | 2 | 0 | 2 | 0 | 2 | 0 | 2 |
|  |  | +/- | 12 | 0 | 12 | 0 | 12 | 0 | 12 |
|  |  | -/- | 6 | 0 | 6 | 0 | 6 | 0 | 6 |
|  | TAM 200 mg/kg (Sigma) | +/+ | 15 | 7 | 8 | 2 | 13 | 7 | 8 |
|  |  | +/- | 21 | 6 | 15 | 0 | 21 | 6 | 15 |
|  |  | -/- | 16 | 4 | 12 | 1 | 15 | 5 | 11 |
| *Esr2* | Vehicle | +/+ | 0 | 0 | 0 | 0 | 0 | 0 | 0 |
|  |  | +/- | 9 | 0 | 9 | 0 | 9 | 0 | 9 |
|  |  | -/- | 7 | 0 | 7 | 0 | 7 | 0 | 7 |
|  | TAM 200 mg/kg (Sigma) | +/+ | 2 | 0 | 2 | 0 | 2 | 0 | 2 |
|  |  | +/- | 22 | 2 | 20 | 0 | 22 | 2 | 20 |
|  |  | -/- | 32 | 4 | 28 | 0 | 32 | 4 | 28 |
